# Lassa fever outcomes and prognostic factors in Nigeria (LASCOPE): a prospective cohort study

**DOI:** 10.1016/S2214-109X(20)30518-0

**Published:** 2021-03-16

**Authors:** Alexandre Duvignaud, Marie Jaspard, Ijeoma Chukwudumebi Etafo, Delphine Gabillard, Béatrice Serra, Chukwuyem Abejegah, Camille le Gal, Abiodun Tolani Abidoye, Mahamadou Doutchi, Sampson Owhin, Benjamin Séri, Jackson Katembo Vihundira, Marion Bérerd-Camara, Justine Schaeffer, Nicolas Danet, Augustin Augier, Ephraim Ogbaini-Emovon, Alex Paddy Salam, Liasu Adeagbo Ahmed, Sophie Duraffour, Peter Horby, Stephan Günther, Akinola Nelson Adedosu, Oladele Oluwafemi Ayodeji, Xavier Anglaret, Denis Malvy, LASCOPE study group, Josephine Funmilola Alabi, Moses Adeniyi Adedokun, Adewale Oladayo Akinpelu, Oyebimpe Ope Oyegunle, Titilola Deborah Sule, Johnson Etafo, Ayoleyi Omowunmi Dede, Macdonald Nonso Onyechi, Moronke Uzuajemeh Ireneh, Olufunke Gbenga-Ayeni, Kehinde Gbemisola Fadiminiyi, Patience Iziegbe Ehigbor, Eric Ouattara, Sophie Karcher, Larissa N'guessan-Koffi, Irmine Ahyi, Elvis Amani, Mamoudou Diabaté, Bertine Siloué, Claire Levy-Marchal, Kader Issaley, Jean-Paul de Bruyne Mushenvula

**Affiliations:** a Inserm 1219, University of Bordeaux, and French National Research Institute for Sustainable Development, Bordeaux, France; b Department of Infectious Diseases and Tropical Medicine, Division of Tropical Medicine and Clinical International Health, University Hospital Centre of Bordeaux, Hôpital Pellegrin, Bordeaux, France; c Programme PAC-CI/ANRS Research Site, University Hospital Centre of Treichville, Abidjan, Côte d'Ivoire; d The Alliance for International Medical Action, Dakar, Senegal; e Lassa Fever Response Team, Infection Control and Research Centre, Federal Medical Centre Owo, Owo, Nigeria; f Viral Hemorrhagic Fever Laboratory, Infection Control and Research Centre, Federal Medical Centre Owo, Owo, Nigeria; g Department of Infectious Diseases, National Hospital Centre of Zinder, Zinder, Niger; h Institute of Lassa Fever Research and Control, Irrua Specialist Teaching Hospital, Irrua, Nigeria; i Centre for Tropical Medicine and Global Health, Nuffield Department of Medicine, University of Oxford, Oxford, UK; j Department of Family Medicine, Federal Medical Centre Owo, Owo, Nigeria; k Department of Virology, Bernhard Nocht Institute for Tropical Medicine, Hamburg, Germany; l German Center for Infection Research, Partner Site Hamburg–Lübeck–Borstel–Riems, Hamburg, Germany

## Abstract

**Background:**

Lassa fever is a viral haemorrhagic fever endemic in parts of west Africa. New treatments are needed to decrease mortality, but pretrial reference data on the disease characteristics are scarce. We aimed to document baseline characteristics and outcomes for patients hospitalised with Lassa fever in Nigeria.

**Methods:**

We did a prospective cohort study (LASCOPE) at the Federal Medical Centre in Owo, Nigeria. All patients admitted with confirmed Lassa fever were invited to participate and asked to give informed consent. Patients of all ages, including newborn infants, were eligible for inclusion, as were pregnant women. All participants received standard supportive care and intravenous ribavirin according to Nigeria Centre for Disease Control guidelines and underwent systematic biological monitoring for 30 days. Patients' characteristics, care received, mortality, and associated factors were recorded using standard WHO forms. We used univariable and multivariable logistic regression models to investigate an association between baseline characteristics and mortality at day 30.

**Findings:**

Between April 5, 2018, and March 15, 2020, 534 patients with confirmed Lassa fever were admitted to hospital, of whom 510 (96%) gave consent and were included in the analysis. The cohort included 258 (51%) male patients, 252 (49%) female patients, 426 (84%) adults, and 84 (16%) children (younger than 18 years). The median time between first symptoms and hospital admission was 8 days (IQR 7–13). At baseline, 176 (38%) of 466 patients had a Lassa fever RT-PCR cycle threshold (Ct) lower than 30. From admission to end of follow-up, 120 (25%) of 484 reached a National Early Warning Score (second version; NEWS2) of 7 or higher, 67 (14%) of 495 reached a Kidney Disease–Improving Global Outcome (KDIGO) stage of 2 or higher, and 41 (8%) of 510 underwent dialysis. All patients received ribavirin for a median of 10 days (IQR 9–13). 62 (12%) patients died (57 [13%] adults and five [6%] children). The median time to death was 3 days (1–6). The baseline factors independently associated with mortality were the following: age 45 years or older (adjusted odds ratio 16·30, 95% CI 5·31–50·30), NEWS2 of 7 or higher (4·79, 1·75–13·10), KDIGO grade 2 or higher (7·52, 2·66–21·20), plasma alanine aminotransferase 3 or more times the upper limit of normal (4·96, 1·69–14·60), and Lassa fever RT-PCR Ct value lower than 30 (4·65, 1·50–14·50).

**Interpretation:**

Our findings comprehensively document clinical and biological characteristics of patients with Lassa fever and their relationship with mortality, providing prospective estimates that could be useful for designing future therapeutic trials. Such trials comparing new Lassa fever treatments to a standard of care should take no more than 15% as the reference mortality rate and consider adopting a combination of mortality and need for dialysis as the primary endpoint.

**Funding:**

Institut National de la Santé et de la Recherche Médicale, University of Oxford, EU, UK Department for International Development, Wellcome Trust, French Ministry of Foreign Affairs, Agence Nationale de Recherches sur le SIDA et les hépatites virales, French National Research Institute for Sustainable Development.

## Introduction

Lassa fever is a viral haemorrhagic fever caused by the Lassa virus. It is endemic in west Africa, where it has an estimated annual burden of 300 000 cases and 5000–10 000 deaths.^1^, ^2^ Lassa virus is mainly transmitted from a zoonotic reservoir to humans,^3^ whereas human-to-human transmission is less frequent and mostly nosocomial.^4^, ^5^ Early symptoms are not very specific, often leading health-care workers to suspect another diagnosis and causing delays in the appropriate management. In severe cases, the course of the disease can lead to multiorgan failure and death.^3^ Most data on the natural history of Lassa fever are retrospective or from before the development of reliable diagnostic tools.^6^, ^7^, ^8^, ^9^ Intravenous ribavirin is the recommended specific treatment despite weak evidence of its efficacy^6^, ^10^ and concerns regarding its toxicity.^10^ No vaccine is available yet. For all these reasons, Lassa fever has been included in the WHO Research and Development Blueprint list of high-priority diseases.

**Research in context**

**Evidence before this study**

Lassa fever is endemic to west Africa, with some reports of exported confirmed cases. Lassa fever is responsible for a disease burden exceeding that of all other viral haemorrhagic fevers except dengue and yellow fever. Prospective cohort evidence is needed on mortality and associated factors under the best standard of care to inform the design of future therapeutic trials. We searched PubMed up to Nov 11, 2020, for publications in English using the following search terms: (“Lassa fever” OR “Lassa virus”) AND (“retrospective study” OR “prospective study” OR “cohort”). Among 19 study reports, only three reported mortality and its associated factors among patients with Lassa fever confirmed by RT-PCR. All three studies were retrospective, and only one study enrolled more than 100 patients.

**Added value of this study**

We monitored a cohort of 510 participants with RT-PCR-confirmed Lassa fever admitted over a 24-month period at the Federal Medical Centre of Owo, Ondo State, Nigeria. Patients received intravenous ribavirin therapy and standardised supportive care free of charge, including fluid resuscitation, antibiotics, antimalarials, blood transfusions, and renal replacement therapy where appropriate. The 30-day mortality was 13% for adults and 6% for children. The median time between symptom onset and admission to the Lassa fever ward was 8 days. 82% of deaths occurred within 7 days of admission. The factors associated with mortality were age, the National Early Warning Score (second version), baseline Lassa RT-PCR cycle threshold value, acute renal failure, and plasma aminotransferase level.

**Implications of all the available evidence**

Lassa fever displays some common characteristics with Ebola virus disease and some striking differences, all of which have implications for the design of future clinical trials. On one hand, as with Ebola, faster diagnosis and management is necessary because most patients arrive late and intra-hospital deaths occur quickly. Additionally, cycle threshold values for Lassa fever RT-PCR appear to be a useful and easy-to-use prognostic tool, for both managing clinical cases and stratifying randomisation for future trials. Furthermore, acute renal failure is a major driver of prognosis and further exploration is needed on carefully balancing recourse to fluid resuscitation, vasopressor or inotropic drugs, and dialysis in this context. On the other hand, mortality from Lassa fever is several times lower than that from Ebola. Because a very large number of participants would be required for a trial to show the efficacy of new drugs in reducing mortality, composite endpoints combining mortality with clinically significant parameters should be considered for future trials.

Several antivirals and immunotherapeutics are promising candidates for treatment of Lassa fever.^11^, ^12^ Before designing drug efficacy trials, it is essential to describe the prospective course of Lassa fever and estimate the mortality rate and associated factors under the best possible standard of care.

In 2018, in the context of an unprecedented seasonal recrudescence of Lassa fever,^13^ we established a Lassa fever ward, organised standardised care, and launched a prospective cohort of patients with Lassa fever at the Federal Medical Centre in Owo (Ondo State, Nigeria).^14^ In this study, we aimed to prospectively study and document the characteristics on admission, care received, and outcomes of patients registered during the first 24 months of this cohort.

## Methods

### Patients and setting

The protocol for the LASCOPE (Lassa fever clinical course and prognostic factors in an epidemic context) cohort has been published previously.^14^ Briefly, the Federal Medical Centre Owo (FMCO) is a tertiary hospital serving a large, semi-urban and rural area in Ondo State, Nigeria. From the start of the study period, all patients admitted to the emergency room and any other department at the FMCO with confirmed Lassa fever were referred to the Lassa fever ward and invited to participate in the cohort. Patients who signed an informed consent form were included in the cohort. Patients of all ages were eligible for inclusion. Pregnant women and newborn infants were also eligible for inclusion. Lassa fever was confirmed by use of the RealStar Lassa Virus *RT*-*PCR* kit 2.0 (Altona Diagnostics, Hamburg, Germany; details presented in appendix p 4).

Informed consent was sought on admission. For underage children (younger than 18 years), the consent of at least one parent or guardian was required. The assent of the child was also sought for children older than 12 years. For adults without the capacity to give informed consent, a relative was asked to consent on behalf of the patient. When the patient regained the capacity to give informed consent, they were asked if they agreed to continue to participate. The study was approved by the Nigerian National Health Research Ethics Committee and the FMCO Research Ethics Committee. Confidentiality was guaranteed by deidentification and restricted access to study documents and databases.

### Follow-up and care of patients

Following the Nigerian Centre for Disease Control guidelines, patients with suspected Lassa fever with a low index of suspicion were not started on ribavirin before RT-PCR confirmation. Patients with confirmed Lassa fever and those without confirmation but with a high index of suspicion (defined in appendix p 3) were started on ribavirin immediately. Different intravenous ribavirin regimens were used depending on age, pregnancy status, and physician's preference (appendix p 3).^15^

Standard supportive care included oral or intravenous administration of fluids and analgesics, oxygen according to the patient's clinical status, antimalarials for patients with confirmed malaria, antibiotics for patients with a suspected bacterial infection, total blood transfusion for patients with severe anaemia, and intermittent haemodialysis for patients with life-threatening acute renal dysfunction. Mechanical ventilation, invasive haemodynamic monitoring, vasopressor drugs, and inotropic drugs were not available.

Patients underwent systematic biological monitoring, including testing for malaria with a rapid diagnostic test (SD BIOLINE Malaria Ag P.f, Abbott, Chicago, IL, USA) or thick blood smear on hospital admission (day 0) and the following tests on day 0, day 5, and day 10 of hospital stay: Lassa virus RT-PCR, full blood count, albuminaemia, creatininaemia, uraemia, plasma electrolytes, aspartate aminotransferase, alanine aminotransferase, and bilirubin. Women of childbearing age underwent a urine pregnancy test at day 0. Additional tests were done whenever it was deemed useful.

The criteria for hospital discharge were absence of fever or any other substantial symptoms and completion of 10 days of ribavirin therapy. Patients meeting the criteria for discharge but still having a positive RT-PCR test for Lassa virus were discharged with a prescription for oral ribavirin for an additional 7 days, as recommended in the Nigerian Centre for Disease Control guidelines (appendix p 3).^15^ Patients who were discharged were asked to attend an outpatient visit 30 days after admission to detect any subacute sequelae requiring specific care.

### Data collection and definitions

We collected demographic characteristics, clinical measures and symptoms, the previously mentioned biological monitoring, care and treatments received, and clinical outcomes. Data were recorded with use of an expanded version of the standard WHO Lassa fever case report form.^14^ Clinical severity was scored with the National Early Warning Score, version 2 (NEWS2).^16^ Acute kidney dysfunction was classified as acute kidney injury or acute kidney failure and staged according to Kidney Disease–Improving Global Outcome (KDIGO) criteria (appendix p 2).^17^, ^18^ Malaria was defined as presentation of signs and symptoms compatible with a malaria episode and the presence of asexual forms of *Plasmodium* sp on a thick blood smear or a positive malaria rapid diagnostic test.

Pregnancy outcomes were categorised as spontaneous miscarriage (spontaneous expulsion of the fetus before 28 weeks of amenorrhoea), intrauterine death (fetal death after 28 weeks of amenorrhoea without fetus expulsion), maternofetal demise (concomitant death of the mother and the fetus), stillbirth (birth of neonate showing no signs of life after 28 weeks of amenorrhoea), and livebirth.^19^

### Statistical analysis

Hospital admission was defined as day 0. We used the Kaplan-Meier method to estimate time to death between day 0 and day 30. We used univariable and multivariable logistic regression models to investigate an association between baseline characteristics and mortality at day 30. All cycle threshold (Ct) values used in the analysis were Ct values for the *GPC* gene. Sex was included a priori in all multivariable models. Age, Ct value, plasma alanine aminotransferase concentration, NEWS2, and KDIGO stage were included in all multivariable models provided they had p<0·05 in univariable analysis. All other variables with less than 10% of missing values and p<0·05 in univariable analysis were included in the initial multivariable model and either kept until the final model or excluded before the final model in a stepwise descending procedure. We did the main analysis with available data. We did a sensitivity analysis including all patients with missing data in a specific category for each variable to explore whether missing data for key variables could influence the final conclusion. Analyses were done with use of SAS, version 9.4.

### Role of the funding source

The funders of the study had no role in study design, data collection, data analysis, data interpretation, or writing of the report. AD, MJ, and DG had access to the raw data. The corresponding author had full access to all of the data and the final responsibility to submit for publication.

## Results

**Figure 1:**
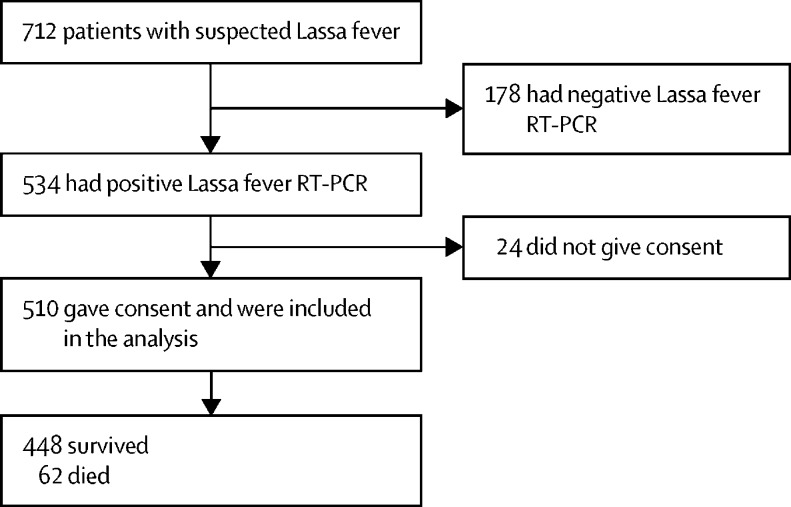
Flow chart of participants

Between April 5, 2018, and March 15, 2020, 712 patients were admitted to the Lassa fever ward with suspected Lassa fever, of whom 178 had negative and 534 had positive Lassa RT-PCR. Of 534 patients with confirmed Lassa fever, 510 (96%) participants signed informed consents and were included in the analysis (figure 1). Of the remaining 24 patients, 15 had impaired consciousness at baseline and died with no relative available of whom consent could be asked and nine declined to take part.

Of the 510 participants, 258 (51%) were male and 252 (49%) female. The median age was 32 years (IQR 21–47), with 84 (16%) participants younger than 18 years and 426 (84%) adults (table 1). 269 (53%) participants came directly from their homes and 241 (47%) were referred from other facilities (82 [16%] from private clinics, 34 [7%] from primary public facilities, and 125 [25%] from secondary or tertiary public facilities). The median time between first symptoms and admission to the Lassa fever ward was 8 days (IQR 7–13). Before admission, 376 (74%) patients had received antimalarials and 14 (3%) ribavirin therapy.

**Table 1:** Baseline and follow-up characteristics

		**Patients with available baseline data (n=510)**	**Baseline**	**Entire follow-up**
**General characteristics**				
Sex		510	..	..
	Male	..	258 (51%)	..
	Female	..	252 (49%)	..
Age, years		510	..	..
	<1	..	4 (1%)	..
	1–4	..	14 (3%)	..
	5–17	..	66 (13%)	..
	18–44	..	277 (54%)	..
	45–59	..	85 (17%)	..
	≥60	..	64 (13%)	..
Number of people in household		498	6 (4–8)	..
Rodents at home or in surrounding area		507	493 (97%)	..
Contact with ill or dead person within 3 weeks of admission		506	63 (12%)	..
Health workers		507	21 (4%)	..
**Vital signs**				
Level of consciousness		508	..	..
	Alert	..	476 (94%)	437 (86%)
	Confusion	..	20 (4%)	13 (3%)
	Voice (reactive to)	..	2 (<1%)	5 (1%)
	Pain (reactive to)	..	6 (1%)	9 (2%)
	Unresponsive	..	4 (1%)	46 (9%)
Heart rate >110 per min		510	73 (14%)	167 (33%)
Systolic arterial pressure <90 mm Hg		492	26 (5%)	107 (22%)
Oxygen saturation <92%		504	34 (7%)	77 (15%)
NEWS2		484	..	..
	0–2	..	199 (41%)	34 (7%)
	3–4	..	130 (27%)	154 (32%)
	5–6	..	90 (19%)	145 (30%)
	≥7	..	65 (13%)	120 (25%)
**Other clinical signs and symptoms**				
Fever (temperature >38·0 °C)		509	389 (76%)	406 (80%)
Headache		508	187 (37%)	220 (43%)
Abdominal pain		509	157 (31%)	191 (38%)
Myalgia		510	101 (20%)	121 (24%)
Chest or retrosternal pain		501	59 (12%)	94 (19%)
Sore throat		508	62 (12%)	69 (14%)
Dizziness		508	127 (25%)	145 (29%)
**Signs of encephalopathy**				
	Seizure	508	12 (2%)	27 (5%)
	Delirium	510	9 (2%)	18 (4%)
	Meningeal syndrome	510	4 (1%)	17 (3%)
	Focal deficiency	510	3 (1%)	6 (1%)
	Aphasia or dysarthria	510	1 (<1%)	2 (<1%)
Impaired hearing or tinnitus		509	2 (<1%)	5 (1%)
Impaired vision		509	2 (<1%)	2 (<1%)
Vomiting		497	190 (38%)	241 (48%)
Watery diarrhoea		508	120 (24%)	148 (29%)
Hiccup		509	1 (<1%)	8 (2%)
Lower limbs oedema		510	10 (2%)	38 (7%)
Facial swelling		509	10 (2%)	17 (3%)
Cough		509	96 (19%)	118 (23%)
Bleeding, any type		510	98 (19%)	174 (34%)
	Macroscopic haematuria	510	27 (5%)	130 (25%)
	Melena	508	21 (4%)	46 (9%)
	Vaginal bleeding*	228	7 (3%)	12 (5%)
	Haematemesis	508	15 (3%)	15 (3%)
	Gingival bleeding	510	5 (1%)	9 (2%)
	Venous puncture point bleeding	509	2 (<1%)	7 (1%)
	Haematochezia	508	6 (1%)	6 (1%)
	Conjunctival bleeding	510	4 (1%)	4 (1%)
	Epistaxis	509	5 (1%)	7 (1%)
	Purpura	510	2 (<1%)	10 (2%)
	Haemoptysis	510	2 (<1%)	2 (<1%)
**Biological measures**				
Lassa virus RT-PCR Ct value†		466	32·0 (27·6–35·2)	..
	≥35·0	..	126 (27%)	..
	30·0–34·9	..	164 (35%)	..
	25·0–29·9	..	108 (23%)	..
	<25·0	..	68 (15%)	..
Haematocrit, %		486	33 (29–38)	28 (24–33)
	<25%	..	50 (10%)	153 (31%)
Platelets, 10^9^ per L		425	200 (118–300)	178 (104–269)
	<80	..	47 (11%)	60 (14%)‡
Leucocytes, 10^9^ cells per L		442	5·8 (3·9–9·3)	7·4 (4·9–11·6)
	>12	..	86 (19%)	105 (24%)
	<4	..	116 (26%)	144 (33%)
Positive malaria diagnostic test§		315	179 (57%)	185 (59%)
Creatininaemia, μmol/L		495	86 (65–115)	92 (73–128)
Uraemia, mmol/L		500	3·5 (2·2–5·5)	3·8 (2·9–6·2)
Acute kidney dysfunction stage		495	..	..
	No dysfunction	..	431 (87%)	410 (83%)
	KDIGO stage 1	..	11 (2%)	18 (4%)
	KDIGO stage 2 (AKI)	..	13 (3%)	10 (2%)
	KDIGO stage 3 (AKF)	..	40 (8%)	57 (12%)
Sodium, mmol/L		487	135 (131–138)	133 (129–136)
	>145	..	23 (5%)	35 (7%)
	<128	..	38 (8%)	84 (17%)
Potassium, mmol/L		482	3·9 (3·6–4·4)	4·2 (3·8–4·7)
	>5·0	..	41 (9%)	73 (15%)
	<3·5	..	99 (21%)	205 (43%)
Total carbon dioxide, mmol/L		479	23 (20–25)	22 (18–24)
	<18	..	75 (16%)	107 (22%)
Albumin, g/L		405	30 (26–33)	29 (24–32)
	<28	..	142 (35%)	171 (42%)
Glycaemia, mmol/L		409	4·8 (4·0–6·1)	4·2 (3·4–5·1)
	<3·0	..	30 (7%)	63 (15%)
AST, U/L¶		408	80 (48–214)	93 (52–232)
	>3 times the upper limit of normal range	..	154 (38%)	166 (41%)
ALT, U/L¶		421	53 (30–107)	62 (35–119)
	>3 times the upper limit of normal range	..	80 (19%)	90 (21%)

Data are n, n (%), or median (IQR). Baseline values were collected on admission to hospital. For entire follow-up, values for vital signs and severity criteria are the poorest condition recorded at any time from baseline to end of follow-up; values for signs and symptoms are those reported at least once from baseline to end of follow-up; and values for biological measures are the poorest value recorded from baseline to end of follow-up. The poorest value for biological parameters was defined as the highest value for leucocytaemia, creatininaemia, uraemia, kalaemia, plasma AST and ALT; and the lowest value for haematocrit, platelet count, natraemia, plasma total carbon dioxide, albuminaemia, and glycaemia. AKF=acute kidney failure. AKI=acute kidney injury. ALT=alanine aminotransferase. AST=aspartate aminotransferase. Ct=cycle threshold. KDIGO=Kidney Disease–Improving Global Outcome. NEWS2=National Early Warning Score, second version.

* Among females aged 12 years or older.

† The 466 (91%) available Ct values were all Ct values for the *GPC* gene; 44 (9%) patients had no available *GPC* gene Ct value, including seven patients with Ct values available for the *L* gene but not for *GPC* and 37 patients with a positive RT-PCR test result but no available Ct value; these patients with no Ct value were included during the first months of the cohort, when Lassa fever molecular diagnostic was not done on site yet and samples were sent to the Irrua Specialist Teaching Hospital laboratory (Irrua, Nigeria), and RT-PCR results were rendered as negative or positive with no Ct values.

‡ Of the 60 patients, two had a platelet count lower than 20 10^9^ per L, 20 had 20–50 10^9^ per L, and 38 had 50–80 10^9^ per L.

§ 315 participants had a malaria diagnostic test done between 7 days before and 4 days after admission; among these, 179 had at least one positive test (thick blood smear or rapid diagnostic test), of whom 40 had a positive thick blood smear test and a negative rapid diagnostic test, 119 had a positive thick blood smear test and no available rapid diagnostic test, 17 had a positive rapid diagnostic test and no available thick blood smear, and three had both tests positive; six additional participants had malaria diagnosed later during their hospital stay (all with a positive rapid diagnostic test, with thick blood smear not done).

¶ The 102 (20%) AST and 89 (17%) ALT missing values were from patients included during the second half of January, 2020, because the biology device used for liver function tests was out of order.

Clinical and biological features of patients on admission and during follow-up are presented in table 1 and the appendix (pp 6–9). The median Ct value for *GPC* in Lassa virus RT-PCR tests at baseline was 32 (IQR 27·6–35·2; appendix p 18). The most common clinical characteristics were fever, pain (headache, myalgia, chest, or abdominal pain), digestive disorders (vomiting or diarrhoea), neurological disorders (dizziness or impaired consciousness) and haemorrhagic signs, of which macroscopic haematuria and melena were the most frequent.

Overall, between baseline and end of follow-up, 120 (25%) of 484 patients reached a NEWS2 of 7 or higher; 67 (14%) of 495 reached a KDIGO stage of 2 or higher; 174 (34%) of 510 had at least one haemorrhagic sign, 73 (14%) of 508 had a consciousness disorder of any grade; 77 (15%) of 504 had oxygen saturation lower than 92%; 153 (31%) of 486 had a haematocrit level lower than 25%; 90 (21%) of 421 had plasma alanine aminotransferase over three times the upper limit of normal; and 171 (42%) of 405 had albuminaemia lower than 28 g/L. Male patients had more frequent acute kidney injury and acute kidney failure and elevated alanine aminotransferase at baseline than did female patients (appendix p 10).

All participants received intravenous ribavirin therapy for a median of 10 days (IQR 9–13; table 2). Ribavirin was started a median of 8 days (7–13) from the onset of symptoms. 92 (18%) participants received oxygen therapy, 158 (31%) had at least one blood transfusion, and 42 (8%) underwent dialysis. Of the 510 participants, 130 still had a positive Lassa virus RT-PCR at day 10, 53 at day 15, 30 at day 20, and 12 at day 25 (appendix p 19). The median time to hospital discharge in patients who survived was 11 days (IQR 10–15).

**Table 2:** Care and treatments

			**Participants**
**Length of hospital stay, days**			
	Time to death, days (n=61)		3 (1–6)
	People who were discharged alive from hospital (n=449)*		11 (10–15)
Admission in the intensive care unit† of the Lassa fever ward			94 (18%)
**Ribavirin therapy**			
	Received		510 (100%)
	Time between first symptoms and first dose, days		8 (7–13)
	Duration of treatment, days		10 (9–13)
**Oxygen**‡			
	Received		92 (18%)
	Maximum output, L/min		6 (5–6)
	Duration, days		3 (2–7)
**Total blood transfusion**§			
	Received		158 (31%)
	Number of units (total blood pints)		2 (2–4)
**Renal replacement therapy (intermittent haemodialysis)**			
	Received		42 (8%)
	Indications¶		
		Fluid overload, no response to diuretics	5 (14%)
		Symptomatic hyperazotaemia	33 (94%)
		Severe acid-base disorder not responding to medical treatment	1 (3%)
	Number of sessions		2 (1–4)
	Outcome		
		Died	23 (56%)
		Survived	18 (44%)
**Antibacterial therapy**			
	Received		459 (90%)
	Duration, days		8 (7–10)
**Antimalarial therapy**‖			
	Received		188 (37%)

Data are n (%) for category variables and median (IQR) for continuous variables.

* One patient died after discharge.

† Intensive care unit refers to a specific area of the Lassa fever ward where patients were more frequently monitored for vital functions; mechanical ventilation, vasopressive drugs, and invasive haemodynamic monitoring were not available.

‡ Missing data for one patient.

§ Missing data for two patients.

¶ Missing data for six patients; four participants had more than one indication for renal replacement therapy: symptomatic hyperazotaemia plus fluid overload not responding to diuretics (n=3) and symptomatic hyperazotaemia plus severe acid-base disorder not responding to medical treatment (n=1).

‖ Antimalarial therapy administered within the Lassa fever ward (treatments before admission are not included).

62 (12%) participants died, including five (6%) of 84 children (four girls and one boy, aged 2 weeks, 4 months, 1 year, 12 years, and 15 years) and 57 (13%) of 426 adults. The median time to death was 3 days (IQR 1–6). Death occurred within 18 days of the onset of symptoms for 48 (81%) of 59 participants with a known date of first symptoms (appendix p 20). Of the 62 deaths, 51 occurred within 7 days of hospital admission, seven between 8 and 19 days, and four more than 20 days after admission (appendix p 21). The four patients who died after 20 days were a 33-year-old man and two women (22 and 45 years) who developed acute kidney failure and underwent dialysis, and a 76-year-old woman with persisting hyponatraemia and severe hepatitis after completing her treatment for Lassa fever (her post-treatment RT-PCR was negative). All but one death occurred in hospital. The participant who died after hospital discharge was a 2-week-old girl at the time of admission. She was discharged at day 12 and died at home 1 week later. Her cause of death was unknown. In univariable analysis, the risk of death increased significantly with increasing age and with the following baseline characteristics: high body-mass index, impaired consciousness, at least one other sign of encephalopathy, low oxygen saturation, accelerated heart rate, watery diarrhoea, at least one sign of bleeding, low platelet count, high leucocyte count, high neutrophil count, high uraemia, high creatininaemia, hypernatraemia, hyperkalaemia, low total carbon dioxide, low albuminaemia, high plasma aspartate aminotransferase or alanine aminotransferase, high plasma bilirubin, high KDIGO grade, high NEWS2 score, low Lassa fever RT-PCR Ct value, and dipstick haematuria (appendix pp 11–15).

We observed no association between mortality and sex, time from first symptoms to hospital admission, time from first symptoms to ribavirin treatment start, a positive malaria test, pregnancy, or any other symptoms or test results (appendix pp 11–15).

**Figure 2:**
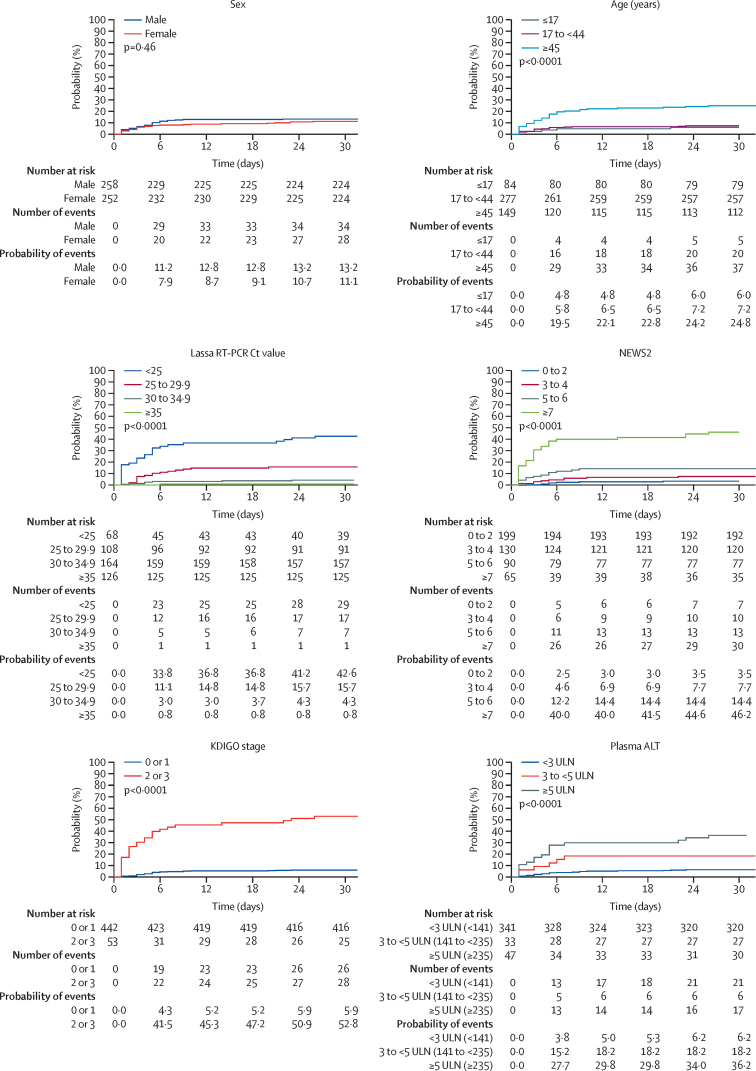
Kaplan-Meier probability of death, according to baseline characteristics ALT=alanine aminotransferase. Ct=cycle threshold. KDIGO=Kidney Disease–Improving Global Outcome. NEWS2=National Early Warning Score, second version. ULN=upper limit of normal range.

In multivariable analysis, age, NEWS2, KDIGO stage, plasma alanine aminotransferase, and RT-PCR Ct value were independently associated with the risk of death (final model, table 3; findings from a sensitivity analysis including patients with missing values are presented in the appendix [p 16]). The probability of death at day 30 was 7·2% for participants younger than 45 years and 24·8% for those aged 45 years or older; 3·5% for participants with baseline NEWS2 of 0–2, 7·7% for those with NEWS2 3–4, 14·4% for those with NEWS2 5–6, and 46·2% for those with NEWS2 of 7 or higher; 5·9% for participants with a baseline KDIGO stage of 0–1 and 52·8% for those with KDIGO stage of 2–3; 6·2% for participants with baseline plasma alanine aminotransferase values lower than 3·0 times the upper limit of normal, 18·2% for those with values 3·0–4·9 times the upper limit of normal, and 36·2% for those with values of 5·0 or more times the upper limit of normal; and 42·6% for participants with a baseline Ct value lower than 25·0, 15·7% for those with Ct 25·0–29·9, 4·3% for those with Ct 30·0–34·9, and 0·8% for those with Ct 35·0 or higher (figure 2). The distribution of baseline Ct values for Lassa fever RT-PCR by age and baseline NEWS2, KDIGO grade, and plasma alanine aminotransferase value is detailed in the appendix (p 17).

**Table 3:** Association between mortality and baseline characteristics, multivariable analysis (n=377)

	**Available data in the univariable analysis**	**Participants who died**	**Univariable**		**Multivariable**	
			Crude odds ratio (95% CI)	p value	Adjusted odds ratio (95% CI)	p value
**Sex**						
Female	252	28 (11%)	1 (ref)	..	1 (ref)	..
Male	258	34 (13%)	1·21 (0·71–2·07)	0·48	1·19 (0·45–3·16)	0·72
**Age, years**						
<45	361	25 (7%)	1 (ref)	..	1 (ref)	..
≥45	149	37 (25%)	4·44 (2·56–7·70)	<0·0001	16·30 (5·31–50·30)	<0·0001
**NEWS2**						
<7	419	30 (7%)	1 (ref)	..	1 (ref)	..
≥7	65	30 (46%)	11·10 (6·02–20·50)	<0·0001	4·79 (1·75–13·10)	0·0023
**Plasma ALT**						
<3 ULN	341	21 (6%)	1 (ref)	..	1 (ref)	..
≥3 ULN	80	23 (29%)	6·15 (3·19–11·80)	<0·0001	4·96 (1·69–14·60)	0·0036
**KDIGO stage**						
<2	442	26 (6%)	1 (ref)	..	1 (ref)	..
≥2	53	28 (53%)	17·90 (9·18–35·00)	<0·0001	7·52 (2·66–21·20)	<0·0001
**Lassa RT-PCR Ct**						
≥30	290	8 (3%)	1 (ref)	..	1 (ref)	..
<30	176	46 (26%)	12·50 (5·72–27·20)	<0·0001	4·65 (1·50–14·50)	0·0078

Data are n (%) unless otherwise specified. ALT=alanine aminotransferase. Ct=cycle threshold (*GPC* gene as a target). KDIGO=Kidney Disease-Improving Global Outcome. NEWS2=National Early Warning Score, second version. ULN=upper limit of normal range.

Of the 172 women of childbearing age, seven (4%) reported a recent miscarriage between the onset of symptoms and hospital admission and 17 (10%) were pregnant on admission. Of these 17 ongoing pregnancies, 12 ended while the participant was in hospital and two ended after hospital discharge (5 days and 5 weeks after discharge). The three remaining women were discharged while still pregnant and their pregnancy outcome was still unknown at the time of the analysis. The 14 documented pregnancy outcomes were six spontaneous miscarriages, one intrauterine death, one maternofetal demise, and six livebirths. The maternofetal demise occurred on day 0. All six live newborn babies tested negative for Lassa fever.

## Discussion

To our knowledge, this is the first prospective study of patients with acute symptomatic Lassa fever confirmed by RT-PCR. Patients received appropriate care in a specific Lassa fever ward, including the possibility of oxygen therapy, blood transfusion, and dialysis. All received intravenous ribavirin in accordance with national and international guidelines. Our findings provide a comprehensive description of clinical and biological characteristics of Lassa fever and their relationship with mortality. More importantly, they provide prospective estimates from a large population for key parameters that will be useful for designing future therapeutic trials.

In this study, the frequency of haemorrhagic signs was higher than that reported at a national level in Nigeria.^13^ The disease displayed a lower mucosal haemorrhaging pattern, in which macroscopic haematuria, melena, and vaginal bleeding were the most frequent manifestations. Thrombopenia was common and moderate.^20^, ^21^ These findings suggest that haemorrhaging might be driven by platelet dysfunction,^20^, ^22^ endothelial dysfunction,^22^ and coagulopathy^21^ rather than thrombocytopenia. We observed a high frequency of hypoalbuminaemia, which might be due to inflammation, digestive loss, renal loss, or vascular leakage.^23^ Kidney dysfunction and its complications (acidosis, hyperkalaemia, and hyperazotaemia) was a dominant feature and a major risk factor for death. However, mortality in patients with severe kidney dysfunction was lower than previously reported,^8^ possibly because of the early access to free-of-charge dialysis. High plasma aminotransferase levels were frequent.^6^, ^8^, ^24^ This was truer for aspartate aminotransferase than for alanine aminotransferase, suggesting that, beyond hepatic tissular damage,^21^, ^25^ some degree of haemolysis or myolysis might occur. Finally, our data show that Lassa fever greatly influences pregnancy outcomes. Seven (4%) women of childbearing age reported miscarriages between the onset of symptoms and hospitalisation. 17 (10%) women were pregnant on hospital admission and eight (57%) of 14 ongoing pregnancies ended with the death of the baby during the hospital stay.^19^, ^26^

All participants received ribavirin therapy. The time between first symptoms and administration of ribavirin was not associated with mortality, contrary to a previous report.^6^ The proportion of patients with a haematocrit level below 25% increased from 10% at baseline to 31% over the entire study follow-up. Ribavirin-induced haemolysis might have occurred even where multiple potential causes of anaemia existed in this context, such as inflammation, massive fluid administration, or haemorrhaging. Most deaths occurred within the first few days. This, together with the 9-day delay between first symptoms and hospital admission, suggests that special efforts should be made to promote earlier diagnosis and faster hospitalisation.

When designing future comparative trials, the expected mortality rate in the reference group will be key for calculating the number of participants needed. In this study, 62 (12%) patients with Lassa fever died, including 57 (13%) of 426 adults and five (6%) of 84 children. These numbers are conservative estimates because some patients who died shortly after admission to hospital could not be asked for informed consent and thus were not included in the analysis. However, we suggest that trials comparing new Lassa fever treatments to a standard of care should take no more than 15% as the reference mortality rate. Given such a mortality rate in the reference group of any trial, large numbers of participants would be required to show that a new treatment can decrease mortality. Therefore, future trial investigators might consider using a composite endpoint, combining mortality with a clinically significant indicator of adverse outcome. We suggest that the worsening of the KDIGO stage and the need for dialysis could be a valuable candidate, because acute kidney failure is a cause of death but renal-associated death can be avoided if the standard of care includes dialysis. Because of the strong association between mortality and baseline Ct value, alanine aminotransferase value, NEWS2, and KDIGO stage, future trial investigators might also consider stratifying the randomisation by one or more of these variables.

Although no prespecified universal cutoff exists for Ct values for Lassa fever RT-PCR assays, we believe that all participants in our study were confirmed cases of Lassa fever. The bell-shaped distribution of the observed Ct values is consistent with a cutoff value at 42 in our setting (appendix p 18). Patients with high Ct values had much lower mortality rates than those with low Ct values, in the same order of magnitude as previously reported in patients with Ebola. This suggests that Ct values are a suitable surrogate for viral load and a useful tool in Lassa fever as well as in Ebola, two diseases where viral load is strongly associated with mortality.^27^

**Our study had several limitations. First, the study was not designed to provide new evidence on the benefit–risk ratio of ribavirin, which is still needed. Second, l**eucocytosis and high neutrophil counts were more frequent in patients who died. However, bacterial cultures were not routinely available. Even if leucocytosis could be due to a stress response in patients with multiorgan failure, it would have been interesting to document the frequency of bacterial morbidity and its potential prognostic value. Third, several other biological variables—such as clotting parameters, inflammatory markers, platelet function, and stigmas of haemolysis or myolysis—were not available, which limits our ability to discuss the pathogenesis underlying some disease features. Finally, we used multivariable logistical regression to analyse the association between mortality and several key characteristics previously shown to be associated with mortality in Ebola virus disease. Our findings are consistent with the hypothesis that there are common clinical and biological features between Ebola and Lassa fever, even if mortality is much higher with Ebola virus disease than with Lassa fever. However, we should point out that odds ratios are not relative risks, and thus the magnitude of the odds ratios we present here does not reflect the respective clinical importance of one variable relative to the others.^28^ Additionally, we did not find any significant association between mortality and other characteristics such as sex or the time from first symptoms to ribavirin treatment start, but the absence of association in a sample of patients does not prove the absence of association in the population.^29^

**In conclusion, this study provides prospective evidence on Lassa fever characteristics under the best possible standard of care in Nigeria. Future studies should explore whether survival could be improved by upgrading the standard of care and whether ribavirin should continue to be part of this standard. Whether the standard of care includes ribavirin or not, future comparative therapeutic trials should actively promote early diagnosis and consider using a composite outcome and stratifying randomisation by baseline Ct value and a clinical or biolog**ical severity score.

## Data sharing

The anonymised individual data and the data dictionary of the LASCOPE study will be made available to other researchers by the coordinating investigator Prof Denis Malvy (denis.malvy@chu-bordeaux.fr) after approval of a methodologically sound proposal and the signing of a data access agreement.
