# Supplementary material for: Lassa fever outcomes and prognostic factors in Nigeria (LASCOPE): a prospective cohort study
Source: Lancet Glob Health. 2021 Mar 16;9(4):e469–78. doi: 10.1016/S2214-109X(20)30518-0 (PMC7970450; doi:10.1016/S2214-109X(20)30518-0)
Supplement: Supplementary appendix [file mmc1.pdf]

THE LANCET
Global Health

Supplementary appendix

This appendix formed part of the original submission and has been peer reviewed. We post it as supplied by the authors.

Supplement to: Duvignaud A, Jaspard M, Etafo IC, et al. Lassa fever outcomes and prognostic factors in Nigeria (LASCOPE): a prospective cohort study. *Lancet Glob Health* 2021; **9:** e469–78.

Supplementary Appendix

This Appendix has been provided by the authors to give readers additional information about their work.

Supplement to: Duvignaud et al, et al. Lassa fever outcomes and prognostic factors: a prospective cohort study in Nigeria (LASCOPE).

Table of Contents

1 Members of the LASCOPE study group not listed as authors ... 2

2 Additional details on the methods ... 2

2.1 Lassa fever case definition ... 2

2.2 Staging of acute kidney injury: the KDIGO classification ... 2

2.3 The National Early Warning Score 2nd version (NEWS2) ... 2

2.4 Ribavirin therapy for Lassa fever (as recommended by NCDC, November 2018) ... 3

2.4.1 McCormick regimen (non-pregnant adults and children) ... 3

2.4.2 Irrua regimen (non-pregnant adults) ... 3

2.4.3 Modified McCormick regimen (pregnant women) ... 3

2.4.4 Additional references ... 3

2.5 Lassa virus RT-PCR ... 4

2.6 Statistical analysis ... 4

3 Supplementary Tables ... 5

3.1 Table S1. Baseline sociodemographic characteristics of Lassa RT-PCR positive patients included in the analysis (N=510) and Lassa RT-PCR negative patients (N=124) ... 5

3.2 Table S2. Baseline and follow-up biological characteristics of Lassa virus RT-PCR positive patients included in the analysis (expanded version) (N=510) ... 6

3.3 Table S3. Baseline characteristics in men and women (N=510) ... 10

3.4 Table S4. Baseline clinical characteristics among Lassa RT-PCR positive patients included in the analysis who died and survived (N=510) ... 11

3.5 Table S5. Baseline biological characteristics among LV RT-PCR positive patients included in the analysis who died and survived (N=510) ... 14

3.6 Table S6. Association between mortality and baseline characteristics, multivariate analysis (N=510): sensitivity analysis including patients with missing values ... 16

3.7 Table S7. Baseline Lassa virus RT-PCR CPC gene Ct value by age, baseline NEWS2, baseline KDIGO grade and baseline plasma ALT value ... 17

4 Supplementary Figures ... 18

4.1 Figure S1. Distribution of the baseline Lassa virus RT-PCR *CPC gene* Ct value in participants included in the analysis (N = 466) ... 18

4.2 Figure S2. Lassa virus RT-PCR result at each testing point in Lassa RT-PCR positive participants included in the analysis (N = 510) ... 19

4.3 Figure S3. Kaplan Meier probability of death among Lassa RT-PCR positive participants with a known date of symptom onset (N=505*) [Time from symptoms onset] 20

4.4 Figure S4. Kaplan Meier probability of death among Lassa virus RT-PCR positive participants included in the analysis (N=510) [Time from admission in the Lassa ward]..21

1 Members of the LASCOPE study group not listed as authors

Federal Medical Centre Owo, Owo, Ondo State, Nigeria

Josephine Funmilola Alabi, Moses Adeniyi Adedokun, Adewale Oladayo Akinpelu, Oyebimpe Ope Oyegunle, Titilola Deborah Sule, Johnson Etafo, Ayoleyi Omowunmi Dede, Macdonald Nonso Onyechi, Moronke Uzuajemeh Ireneh, Olufunke Gbenga-Ayeni, Kehinde Gbemisola Fadiminiyi, Patience Iziegbe Ehigbor.

Inserm 1219, Univ. Bordeaux, IRD, Bordeaux, France

Eric Ouattara, Sophie Karcher.

Programme PAC-CI/ANRS Research Site, CHU de Treichville, Abidjan, Côte d'Ivoire

Larissa N'guessan-Koffi, Irmine Ahyi, Elvis Amani, Mamoudou Diabaté, Bertine Siloué.

The Alliance for International Medical Action, Dakar, Senegal

Claire Levy-Marchal, Kader Issaley, Jean-Paul de Bruyne Mushenvula.

2 Additional details on the methods

2.1 Lassa fever case definition

(From: National guidelines for the management of LF, Nigerian Centre for Disease Control, 2018 version)[1]
Suspected LF: history of fever (body temperature measured above 38°C more than 24 hours without the use of anti-pyretics) beginning 3 to 21 days prior to presentation, with one or more of the following conditions: abdominal pain, vomiting, diarrhoea, sore throat, myalgia, generalized body weakness, abnormal bleeding (mucosal bleeding, punctures sites bleeding, uncontrolled intra operational and/or immediate post operational bleeding).

OR

Neonates (with or without signs and symptoms) from women infected by Lassa virus.

Any of the following scenarios reinforce the index of suspicion:

a. Absence of response to standard anti-malaria treatment and treatment for other common infectious causes of fever within 48-72 hours.
b. History of recent contact with a probable or confirmed case of LF within 21 days of onset of fever.
c. History of recent travel to high risk/burden area of LF within 21 days of onset of fever.
d. Contact with body fluids or tissues of a dead patient with a febrile illness, symptoms and signs highly suggestive of LF leading to death within 21 days of onset of fever.

Confirmed LF: any criteria for suspected LF AND positive Lassa RT-PCR from any body fluid.

2.2 Staging of acute kidney injury: the KDIGO classification

The Kidney Disease | Improving Global Outcome consortium is the global non for profit organization developing and implementing evidence-based clinical practice guidelines for the management of kidney diseases. In 2012, the members of the consortium released clinical practice guidelines for acute kidney injury (AKI) in which they proposed a revision of the previously existing classifications. This new grading system, so-called the KDIGO classification [2,3], relies on serum creatinine and urine output and distinguishes three stages of AKI from 1 to 3. As the measurement of urine output in patients hospitalized in an isolation unit is difficult, especially in those not having a urinary catheter, we made the decision to grade AKI according to the KDIGO classification by using the sole serum creatinine value.

2.3 The National Early Warning Score 2nd version (NEWS2)

The National Early Warning Score[4] is a clinical severity scoring system developed by the Royal College of Physicians to help health care workers with assessing the risk of clinical deterioration and adapt the level of care needed in hospitalised patients with acute diseases, including infectious diseases. The first version, released in 2012, was updated in 2017. The NEWS is based on a simple aggregate scoring system in which a score is allocated to six physiological parameters recorded in routine practice:

1. Respiration rate
2. Oxygen saturation +/- the need for supplemental oxygen
3. Systolic blood pressure
4. Pulse rate
5. Level of consciousness or new confusion according to the ACPVU classification (A: Alert; C: new Confusion; V: reactive to Voice; P: reactive to Pain; U: totally Unreactive)
6. Temperature

Different thresholds have been proposed for this score. They share a common definition of the class at highest risk and level of care needed corresponding to an aggregate score superior or equal to 7. In this work, we defined four classes of risk as follows:

- Low risk for a score from 0 to 2
- Low medium risk for a score from 3 to 4
- Medium risk for a score from 5 to 6
- High risk for a score superior or equal to 7

2.4 Ribavirin therapy for Lassa fever (as recommended by NCDC, November 2018)

The recommended treatment for Lassa fever is ribavirin. In Nigeria, national guidelines for the management of Lassa fever are issued by the Nigeria Centre for Disease Control (NCDC). In their November 2018 update[1], they propose several intravenous ribavirin treatment regimens. In adults, physicians are offered the choice between the “McCormick” regimen, derived from the one historically used in the clinical trial conducted by McCormick and colleagues in Sierra Leone in the 1980s[5], and the “Irrua” regimen, developed at the Irrua Specialist Teaching Hospital (ISTH) situated in Edo State, Nigeria. The latter has the advantage of a lesser number of infusions, leading to a reduced workload for health care workers, and a reduced cumulated dose, thereby sparing ribavirin. In pregnant women, the so-called “modified McCormick regimen”, also developed at ISTH, is recommended by NCDC’s guidelines; it combines the high loading dose used in the “Irrua” regimen and the maintenance dose used in the “McCormick” regimen. “Irrua” and “modified McCormick” regimens have never been evaluated in a clinical trial. Lastly, the “McCormick” regimen is the one recommended to treat Lassa fever in children. Those regimens are detailed hereafter.

Finally, the NCDC guidelines for the management of Lassa fever also recommends that patients who meet the criteria for discharge while still having a positive Lassa RT-PCR should be discharged with a prescription for oral ribavirin for seven more days.

2.4.1 McCormick regimen (non-pregnant adults and children)

Period	Dose	Frequency
Loading dose (day 0)	33 mg/kg (maximum 2640 mg)	Stat
Maintenance dose phase 1 (day 0 to 3)	16 mg/kg (maximum 1280 mg)	6 hourly
Maintenance dose phase 1 (day 4 to 9)	8 mg/kg (maximum 640 mg)	8 hourly

2.4.2 Irrua regimen (non-pregnant adults)

Period	Dose	Frequency
Loading dose (day 0)	100 mg/kg (maximum 7000 mg)	In two divided doses : 2/3 given stat and 1/3 given 8 hours later
Maintenance dose phase 1 (day 1 to 6)	25 mg/kg (maximum 1280 mg)	Daily
Maintenance dose phase 1 (day 7 to 9)	12.5 mg/kg (maximum 640 mg)	Daily

2.4.3 Modified McCormick regimen (pregnant women)

Period	Dose	Frequency
Loading dose (day 0)	100 mg/kg (maximum 7000 mg)	In two divided doses : 2/3 given stat and 1/3 given 8 hours later
Maintenance dose phase 1 (day 1 to 4)	16 mg/kg (maximum 1280 mg)	6 hourly
Maintenance dose phase 1 (day 5 to 9)	8 mg/kg (maximum 640 mg)	8 hourly

2.4.4 Additional references

1 Nigeria Centre for Disease Control. National Guidelines for Lassa Fever Case Management. Nigeria Centre for Disease Control. 2018; published online Nov. https://ncdc.gov.ng/themes/common/docs/protocols/92_1547068532.pdf (accessed Feb 20, 2019).

2 Khwaja A. KDIGO Clinical Practice Guidelines for Acute Kidney Injury. *NEC* 2012; **120**: c179–84.

3 Kellum JA, Lameire N, KDIGO AKI Guideline Work Group. Diagnosis, evaluation, and management of acute kidney injury: a KDIGO summary (Part 1). *Crit Care* 2013; **17**: 204.

4 National Early Warning Score (NEWS) 2. RCP London. 2017; published online Dec 19. https://www.rcplondon.ac.uk/projects/outputs/national-early-warning-score-news-2 (accessed July 1, 2020).

5 McCormick JB, King IJ, Webb PA, *et al.* Lassa fever. Effective therapy with ribavirin. *N Engl J Med* 1986; **314**: 20–6.

2.5 Lassa virus RT-PCR

In March 2019, Lassa fever molecular diagnosis capacities were set up at FMC Owo thanks to the support of the Irrua Specialist Teaching Hospital (ISTH) laboratory, NCDC, WHO, the Pandora network and the Bernhard Nocht Institute of Tropical Medicine. During the first six months all samples analyzed at the FMC Owo laboratory were double-checked at the ISTH laboratory.

The Lassa fever diagnosis was made using the Altona RealStar Lassa 2.0™ kit (https://www.altona-diagnostics.com/en/products/reagents-140/reagents/realstar-real-time-pcr-reagents/realstar-lassavirus-rt-pcr-kit-ce.html). The kit includes two separate reactions for GPC and L viral genes, each running for 45 cycles. The kit also includes negative and positive controls. The negative control allows the detection of non-specific amplification and cross-contaminations. One positive control allows the detection of amplification inhibition. Two additional positive controls using synthetic RNA mimicking the two target viral genes (GCP and L genes) serve as a reference for positive reactions. For a run to be valid, the Ct value of these positive controls needs to be between 30 and 32.

There is no pre-specified universal cut-off for Ct values for real-time RT-PCR assays. The ISTH laboratory, a long-standing reference laboratory for Lassa Fever diagnosis in Nigeria, have set the cut-off values at 42 cycles locally. The FMCO Lassa Fever laboratory was set up by and uses the same workflow as the ISTH laboratory. This includes checking the shape of the amplification curves to ensure that positive results are true positives, not artefactual. Every sample for which there is a doubt regarding the amplification curve is tested twice.

2.6 Statistical analysis

We decided *a priori* that :

- Sex would be included in all multivariable models, regardless of the result of the univariable analysis.
- Age, Ct value, transaminases and renal function would be included in the initial multivariable model provided they had a $p<0{\cdot}05$ in univariable analysis; and would then remain in all steps of the descending procedure. This was because we hypothesized that :
  - Old age, high viral load, hepatic and/or tissular damage and renal function impairment could be associated with mortality in Lassa fever for the same reasons as in Ebola virus disease.
  - Ct value would be a valuable proxy for viral load
  - Transaminases values would reflect hepatic and/or tissular damage
- All other variables with less than 10% missing values would be included in the initial multivariable model if they had a $p<0{\cdot}05$ in univariable analysis; and would be removed during the descending procedure if their p became $>0{\cdot}05$. We had no particular hypothesis regarding these variables.

3 Supplementary Tables

3.1 Table S1. Baseline sociodemographic characteristics of Lassa RT-PCR positive patients included in the analysis (N=510) and Lassa RT-PCR negative patients (N=124)

	**Lassa RT-PCR positive (N = 510)**	**Lassa RT-PCR negative (N = 124)**	***p* ***
Age, years, median [IQR]	32 [21 – 47]	32 [19 – 46]	0.60
Male sex, n (%)	258 (50.6)	66 (53.2)	0.60
Education ≥ secondary school, n (%) (N = 617)	348 (70.2)	89 (73.6)	0.46
Health workers, n (%) (N = 629)	21 (4.1)	8 (6.6)	0.36
Housing location, n (%) (N = 630)			**0.01**
Urban	51 (10.1)	22 (17.7)	
Peri-urban	286 (56.5)	54 (43.5)	
Rural	169 (33.4)	48 (38.7)	
Number of people in household, median [IQR] (N = 617)	6 [4 – 8]	5 [4 – 8]	0.50
Number of children under 15 in household, median [IQR] (N = 617)	2 [1 – 4]	2 [0 – 4]	0.65
Contact with an ill or dead person (within 3 weeks before symptoms onset), n (%) (N = 629)	63 (12.5)	24 (19.5)	**0.04**

*Kruskal-Wallis test for quantitative variables; Chi square test or Fisher exact test for qualitative variables

n=number; %=columns percentage; IQR=interquartile range

3.2 Table S2. Baseline and follow-up biological characteristics of Lassa virus RT-PCR positive patients included in the analysis (expanded version) (N=510).

Biological findings	On admission		Poorest value*		Last available value*	
	Median [IQR]	n (%)	Median [IQR]	n (%)	Median [IQR]	n (%)
Haemoglobin (g/dL) (N = 457)	11.8 [10.3 – 13.1]		10.3 [8.7 – 12.1]		10.9 [9.5 – 12.4]	
< 8.0		26 (5.7)		75 (16.4)		36 (8.1)
Haematocrit (%) (N = 486)	33 [29 – 38]		28 [24 – 33]		30 [27 – 34]	
< 25		50 (10.3)		153 (31.5)		84 (17.5)
Platelets (G/L) (N = 425)	200 [118 – 300]		178 [104 – 269]		255 [173 – 348]	
< 80		47 (11.1)		60 (14.1)		24 (5.8)
< 50		12 (2.8)		22 (5.2)		8 (1.9)
Leucocytes (G/L) (N = 442)	5.8 [3.9 – 9.3]		7.4 [4.9 – 11.6]		5.9 [4.4 – 8.7]	
> 12		86 (19.5)		105 (23.8)		43 (10.0)
< 4		116 (26.2)		144 (32.6)		75 (17.4)
Lymphocytes (G/L) (N = 422)	2.0 [1.4 – 3.3]		1.6 [1.2 – 2.4]		1.9 [1.4 – 2.7]	
< 1.5		125 (29.6)		178 (42.2)		113 (27.6)
< 0.8		14 (3.3)		24 (5.7)		8 (2.0)
Neutrophils (G/L) (N = 416)	3.1 [1.9 – 5.5]		4.0 [2.5 – 7.0]		3.1 [2.1 – 5.0]	
> 7.5		68 (16.3)		96 (23.1)		49 (12.3)
< 0.8		14 (3.4)		20 (4.8)		8 (2.0)
Positive malaria diagnostic test (N = 315)**		179 (56.8)		185 (57.6)		-
Urea (mmol/L) (N = 500)	3.5 [2.2 – 5.5]		3.8 [2.9 – 6.2]		3.1 [2.3 – 4.4]	
> 8		78 (15.6)		97 (19.4)		59 (11.9)
> 20		27 (5.4)		47 (9.4)		25 (5.1)
Creatinine (µmol/L) (N = 495)	86 [65 – 115]		92 [73 – 128]		73 [56 – 95]	
> 106		139 (28.1)		174 (35.2)		97 (19.6)

Table S2 (continuation)

Biological findings	On admission		Poorest value*		Last available value*	
	Median [IQR]	n (%)	Median [IQR]	n (%)	Median [IQR]	n (%)
Acute kidney dysfunction (N = 495)						
No dysfunction		431 (87.1)		410 (82.8)		442 (89.3)
KDIGO 1		11 (2.2)		18 (3.6)		10 (2.0)
KDIGO 2 (AKI)***		13 (2.6)		10 (2.0)		8 (1.6)
KDIGO 3 (AKF)***		40 (8.1)		57 (11.5)		35 (7.1)
Proteinuria ≥ 2 ++ (N=221)		48 (21.7)		72 (32.6)		56 (25.5)
Haematuria ≥ 2 ++ (N=220)		50 (22.7)		67 (30.5)		41 (18.7)
Natraemia (mmol/L) (N = 487)	135 [131 – 138]		133 [129 – 136]		136 [133 – 140]	
> 145		23 (4.7)		35 (7.2)		15 (3.1)
< 128		38 (7.8)		84 (17.2)		34 (7.1)
Kalaemia (mmol/L) (N = 482)	3.9 [3.6 – 4.4]		4.2 [3.8 – 4.7]		3.9 [3.4 – 4.3]	
> 5.0		41 (8.5)		73 (15.1)		32 (6.7)
< 3.5		99 (20.5)		205 (42.5)		128 (26.9)
< 3.0		24 (5.0)		77 (16.0)		33 (6.9)
Chloride (mmol/L) (N = 488)	102 [99 – 106]		105 [102 – 108]		103 [100 – 106]	
> 108		58 (11.9)		110 (22.5)		59 (12.2)
< 98		84 (17.2)		143 (29.3)		61 (12.6)
Calcium corrected from albumin (mmol/L)**** (N=397)	2.4 [2.4 – 2.5]		2.5 [2.4 – 2.6]		2.5 [2.3 – 2.6]	
> 2.60		44 (11.1)		85 (21.3)		64 (16.5)
< 2.00		9 (2.3)		31 (7.7)		16 (4.1)
tCO2 (mmol/L) (N = 479)	23 [20 – 25]		22 [18 – 24]		24 [21 – 26]	
< 18		75 (15.7)		107 (22.3)		40 (8.4)
< 15		26 (5.4)		50 (10.4)		17 (3.6)
< 12		8 (1.7)		24 (5.0)		10 (2.1)

Table S2 (continuation)

Biological findings	On admission		Poorest value *		Last available value*	
	Median [IQR]	n (%)	Median [IQR]	n (%)	Median [IQR]	n (%)
Albumin (g/L) (N = 405)	30 [26 – 33]		29 [24 – 32]		32 [27 – 35]	
< 35		341 (84.2)		361 (89.1)		295 (73.0)
< 28		142 (35.1)		171 (42.2)		110 (27.2)
< 25		79 (19.5)		103 (25.4)		68 (16.8)
Glycaemia (mmol/L) (N = 409)	4.8 [4.0 – 6.1]		4.2 [3.4 – 5.1]		4.8 [4.0 – 6.1]	
< 4.0		110 (26.9)		186 (45.5)		108 (27.4)
< 3.0		30 (7.3)		63 (15.4)		34 (8.6)
AST (U/L) (N = 408)	80 [48 – 214]		93 [52 – 232]		54 [39 – 92]	
> 3 x ULN		154 (37.7)		166 (40.7)		74 (18.6)
> 5 x ULN		114 (27.9)		122 (29.9)		46 (11.6)
ALT (U/L) (N = 421)	53 [30 – 107]		62 [35 – 119]		39 [26 – 67]	
> 3 x ULN		80 (19.0)		90 (21.4)		35 (8.6)
> 5 x ULN		47 (11.2)		52 (12.4)		18 (4.4)
AST or ALT > 3 x ULN (N = 421)		169 (40.1)		181 (43.0)		81 (20.0)
Alkaline Phosphatase (N=420)	71 [53 – 122]		67 [50 – 108]		77 [57 – 133]	
Total bilirubin (µmol/L) (N = 413)	10 [8 – 14]		14 [10 – 20]		12 [9 – 17]	
> 27.4		26 (6.3)		52 (12.6)		29 (7.3)
> 50		13 (3.1)		16 (3.9)		7 (1.8)
Lassa RT-PCR Ct value‡ (N = 466)	32.0 [27.6 – 35.2]					
≥ 35		126 (27.0)				
30 to 34.9		164 (35.2)				
25 to 29 .9		108 (23.2)				
< 25		68 (14.6)				

Footnotes to table S2

n=number; %=columns percentage; IQR=interquartile range

g/dL: gram per deciliter; G/L: giga per liter; mmol/L: millimole per liter; µmol/L: micromole per liter; g/L: gram per liter; ULN: Upper Limit of Normal range.

* Poorest value: poorest value recorded at any time between admission and end of follow-up. The poorest biological value was defined as: (i) the highest value for leucocytes, neutrophils, uraemia, creatininemia, proteinuria, haematuria, kalaemia, chloride, calcium corrected from albumin, AST, ALT, alkaline phosphatase, total bilirubin; (ii) the lowest value for haemoglobin, haematocrit, platelets, lymphocytes, natraemia, tCO2, albuminaemia and glycaemia.

**315 participants had a malaria diagnostic test performed between 7 days before and 4 days after admission. Among these 315 participants, 179 had at least one test positive (TBS: thick blood smear; RDT: rapid diagnostic test) of whom: 40 had a positive TBS and a negative RDT; 119 had a positive TBS and a RDT not available; 17 had a positive RDT and a TBS not available; and 3 had both positive TBS and RDT. 6 additional participants had malaria diagnosed later during their hospital stay (all with a positive RDT and TBS not performed).

***AKI: Acute Kidney Injury; AKF: Acute Kidney Failure.

****Calcium corrected from albumin = Calcemia + 1 – (0.025*Albumin).

‡For the GPC gene target of the Altona Realstar Lassa RT-PCR kit 2.0®.

3.3 Table S3. Baseline characteristics in men and women (N=510)

Characteristics on admission	Men (N=258)	Women (N=252)	*P**
Age (years)			
< 1	2 (0.8)	2 (0.8)	0.99
1 to 11	25 (9.7)	21 (8.3)	
12 to 17	16 (6.2)	18 (7.1)	
18 to 44	138 (53.5)	139 (55.2)	
45 to 59	44 (17.1)	41 (16.3)	
≥ 60	33 (12.8)	31 (12.3)	
Time 1st symptom to admission (days) (N=505)	8 [6 – 13]	9 [7 – 13]	0.63
Level of consciousness (ACVPU classification) (N=508)			0.51
Alert	242 (93.8)	234 (93.6)	
Confusion	9(3.5)	11 (4.4)	
Voice (reactive to)	0 (0)	2 (0.8)	
Pain (reactive to)	4(1.6)	2 (0.8)	
Unresponsive	3 (1.2)	1 (0.4)	
SpO2 (N=504)			0.72
≥ 92 %	242 (93.8)	228 (92.7)	
< 92 %	16 (6.2)	18 (7.3)	
NEWS2** (N=484)			0.57
0 to 2 (low risk)	98 (40.2)	101 (42.1)	
3 or 4 (low medium risk)	67 (27.5)	63 (26.3)	
5 or 6 (medium risk)	50 (20.5)	40 (16.7)	
≥ 7 (high risk)	29 (11.9)	36 (15.0)	
Ct value† (cycles) (N=466)			0.53
≥ 35	70 (29.9)	56 (24.1)	
30 to 34.9	77 (32.9)	87 (37.5)	
25 to 29.9	53 (22.6)	55 (23.7)	
< 25	34 (14.5)	34 (14.7)	
Acute kidney injury or failure (N=495)			**0.0002**
No dysfunction	200 (80.6)	231 (93.5)	
KDIGO 1	8 (3.2)	3 (1.2)	
KDIGO 2	11 (4.4)	2 (0.8)	
KDIGO 3	29 (11.7)	11 (4.5)	
AST (N=408)			0.15
≤ 3 ULN‡	119 (58.6)	135 (65.9)	
> 3 ULN	84 (41.4)	70 (34.1)	
ALT (N=421)			**0.02**
≤ 3 ULN	163 (76.5)	178 (85.6)	
> 3 ULN	50 (23.5)	30 (14.4)	

Data are number (column percentages) for categorical variables, and median [Interquartile range] for continuous variables.

*Kruskal-Wallis test for quantitative variables, Chi square test or Fisher exact test for qualitative variables; **NEWS2 score: National Early Warning Score 2nd version; †For the GPC gene target of the Altona Realstar Lassa kit 2.0® based RT-PCR; ‡ULN: Upper Limit of Normal range.

3.4 Table S4. Baseline clinical characteristics among Lassa RT-PCR positive patients included in the analysis who died and survived (N=510)

	**Survived (N=448)**	**Died (N=62)**	***p***
Age (years)			**<0.0001**
< 1	2 (50.0)	2 (50.0)	
1 to 11	45 (97.8)	1 (2.2)	
12 to 17	32 (94.1)	2 (5.9)	
18 to 44	257 (92.8)	20 (7.2)	
45 to 59	67 (78.8)	18 (21.2)	
≥ 60	45 (70.3)	19 (29.7)	
Sex			0.5
Female	224 (88.9)	28 (11.1)	
Male	224 (86.8)	34 (13.2)	
Body Mass Index (N = 374)			**0.04**
Body Mass Index < 30 kg/m²	321 (95.8)	14 (4.2)	
Body Mass Index ≥ 30 kg/m²	34 (87.2)	5 (12.8)	
Pregnancy			
Women of childbearing age (N = 172)	160 (93.0)	12 (7.0)	
Ongoing pregnancy on admission	15 (93.8)	1 (6.3)	1.0
Time 1st symptom to 1st ribavirin dose (days) (N = 505)	8 (7-13)	9 (7-14)	
< 7 days	111 (88.8)	14 (11.2)	0.79
≥ 7 days	334 (87.9)	46 (12.1)	
**Vital parameters**			
Level of consciousness (ACVPU classification) (N = 508)			**<0.0001**
Alert	436 (91.6)	40 (8.4)	
Confusion	7 (35.0)	13 (65.0)	
Voice (reactive to)	1 (50.0)	1 (50.0)	
Pain (reactive to)	2 (33.3)	4 (66.7)	
Unresponsive	0	4 (100)	
Heart rate			**<0.0001**
≤ 110 per minute	396 (90.6)	41 (9.4)	
> 110 per minute	52 (71.2)	21 (28.8)	
Systolic Arterial Pressure (N = 492)			0.23
≥ 90 mmHg	411 (88.2)	55 (11.8)	
< 90 mmHg	21 (80.8)	5 (19.2)	
Mean Arterial Pressure (N = 492)			0.53
≥ 65 mmHg	411 (88.0)	56 (12.0)	
< 65 mmHg	21 (84.0)	4 (16.0)	
SpO2 (N = 504)			**<0.0001**
≥ 92 %	429 (91.3)	41 (8.7)	
< 92 %	15 (44.1)	19 (55.9)	
NEWS2† (N = 484)			**<0.0001**
0 to 2 (low risk)	192 (96.5)	7 (3.5)	
3 or 4 (low medium risk)	120 (92.3)	10 (7.7)	
5 or 6 (medium risk)	77 (85.6)	13 (14.4)	
≥ 7 (high risk)	35 (53.8)	30 (46.2)	

Table S4(continued). Baseline clinical characteristics among Lassa RT-PCR positive patients included in the analysis who died and survived (N=510)

	Survived (N=448)	Died (N=62)	*p*
**Other signs or symptoms**			
Fever (measured temperature > 38.0°C) (N = 509)			0.34
No	109 (90.8)	11 (9.2)	
Yes	338 (86.9)	51 (13.1)	
Headache (N=508)			**0.0001**
No	270 (84.1)	51 (15.9)	
Yes	178 (95.2)	9 (4.8)	
Dizziness/Vertigo (N = 508)			0.34
No	339 (89.0)	42 (11.0)	
Yes	109 (85.8)	18 (14.2)	
Signs of encephalopathy (other than impaired consciousness) ‡			**<0.0001**
No	435 (89.9)	49 (10.1)	
Yes	13 (50.0)	13 (50.0)	
Impaired hearing or tinnitus (N = 509)			1.0
No	446 (88.0)	61 (12.0)	
Yes	2 (100)	0 (0)	
Sore throat (N=508)			0.06
No	397 (89.0)	49 (11.0)	
Yes	50 (80.6)	12 (19.4)	
Facial swelling (N=509)			0.35
No	439 (88.0)	60 (12.0)	
Yes	8 (80.0)	2 (20.0)	
Cough (N = 509)			0.49
No	365 (88.4)	48 (11.6)	
Yes	82 (85.4)	14 (14.6)	
Chest pain/Retrosternal pain (N=501)			0.28
No	388 (87.8)	54 (12.2)	
Yes	5.5 (93.2)	4 (6.8)	
Abdominal pain (N = 509)			**0.02**
No	304 (86.4)	48 (13.6)	
Yes	144 (91.7)	13 (8.3)	
Vomiting (N=497)			1.0
No	270 (87.9)	37 (12.1)	
Yes	167 (87.9)	23 (12.1)	
Hiccup (N = 509)			0.12
No	448 (88.2)	60 (11.8)	
Yes	0	1 (100)	

Table S4 (continued). Baseline clinical characteristics among Lassa RT-PCR positive patients included in the analysis who died and survived (N=510)

Characteristics on admission	Survived (N=448)	Died (N=62)	*p*
Watery diarrhoea (N=508)			**0.0002**
No	353 (91.0)	35 (9.0)	
Yes	93 (77.5)	27 (22.5)	
Lower limbs oedema			0.35
No	440 (88.0)	60 (12.0)	
Yes	8 (80)	2 (20)	
Any type of bleeding			**<0.0001**
No	381 (92.5)	31 (7.5)	
Yes	67 (68.4)	31 (31.6)	

Data are numbers and rows percentages.

‡Signs of encephalopathy (other than impaired consciousness): seizure, delirium, meningeal syndrome, focal deficiency, aphasia, dysarthria.

3.5 Table S5. Baseline biological characteristics among LV RT-PCR positive patients included in the analysis who died and survived (N=510)

Characteristics on admission	Survived (N=448)	Died (N=62)	*p*
Haemoglobin (g/dL) (N = 457)			0.20
≥ 8.0	384 (89.1)	47 (10.9)	
< 8.0	21 (80.8)	5 (19.2)	
Haematocrit (%) (N = 486)			0.35
≥ 25.0	387 (88.8)	49 (11.2)	
< 25	42 (84.0)	8 (16.0)	
Platelets (G/L) (N = 425)			**0.048**
≥ 80	339 (89.7)	39(10.3)	
< 80	37 (78.7)	10 (21.3)	
Leucocytes (G/L) (N = 442)			**<0.0001**
≤ 12	332 (93.3)	24 (6.7)	
> 12	60 (69.8)	26 (30.2)	
Neutrophils (G/L) (N = 416)			**<0.0001**
≤ 7.5	320 (92.0)	28 (8.0)	
> 7.5	47 (69.1)	21 (30.9)	
Positive malaria diagnostic test** (N=315)			0.73
No	121 (89.0)	15 (11.0)	
Yes	157 (87.7)	22 (12.3)	
Urea (mmol/L) (N = 500)			**<0.0001**
≤ 8	403 (95.5)	19 (4.5)	
> 8	38 (48.7)	40 (51.3)	
Creatinine (µmol/L) (N = 495)			**<0.0001**
≤ 106	343 (96.3)	13 (3.7)	
> 106	98 (70.5)	41 (29.5)	
Acute kidney injury or failure (N = 495)			**<0.0001**
No dysfunction	406 (94.2)	25 (5.8)	
KDIGO 1	10 (90.9)	1 (9.1)	
KDIGO 2	9 (69.2)	4 (30.8)	
KDIGO 3	16 (40.0)	24 (60.0)	
Proteinuria (N=221)			0.51
< 2 ++	147 (85.0)	26 (15.0)	
≥ 2 ++	39 (81.3)	9 (18.8)	
Haematuria (N=220)			**0.001**
< 2 ++	151 (88.8)	19 (11.2)	
≥ 2 ++	34 (68.0)	16 (32.0)	
Sodium (mmol/L) (N = 487)			**0.0004**
≤ 145	417 (89.9)	47 (10.1)	
> 145	14 (60.9)	9 (39.1)	
Potassium (mmol/L) (N = 482)			**<0.0001**
≤ 5.0	407 (92.3)	34 (7.7)	
> 5.0	24 (58.5)	17 (41.5)	
Chloride (mmol/L) (N=488)			**<0.0001**
≤ 108	390 (90.7)	40 (9.3)	
> 108	41 (70.7)	17 (29.3)	

Table S5 (continued). Baseline biological characteristics among LV RT-PCR positive patients included in the analysis who died and survived (N=510)

**Characteristics on admission**	**Survived (N=448)**	**Died (N=62)**	**P value***
Calcium corrected from albumin (mmol/L) (N = 397)			0.06
≥ 2.0	348 (89.7)	40 (10.3)	
< 2.0	6 (66.7)	3 (33.3)	
tCO2 (mmol/L) (N = 479)			**<0.0001**
≥ 18	369 (91.3)	35 (8.7)	
< 18	54 (72.0)	21 (28.0)	
Albumin (g/L) (N = 405)			**<0.0001**
≥ 28	248 (94.3)	15 (5.7)	
< 28	113 (79.6)	29 (20.4)	
Glycaemia (mmol/L) (N=409)			
≥ 3	338 (89.2)	41 (10.8)	1.0
< 3	27 (90.0)	3 (10.0)	
ASAT (N = 408)			**<0.0001**
≤ 3 ULN†	248 (97.6)	6 (2.4)	
> 3 ULN	127 (82.5)	27 (17.5)	
ALAT (N = 421)			**<0.0001**
≤ 3 ULN	320 (93.8)	21 (6.2)	
> 3 ULN	57 (71.3)	23 (28.8)	
Bilirubin (N = 413)			**0.024**
≤ 50.0 mmol/L	366 (91.5)	34 (8.5)	
> 50.0 mmol/L	9 (69.2)	4 (30.8)	
Lassa RT-PCR Ct value (cycles) ‡ (N = 466)			**<0.0001**
≥ 35	125 (99.2)	1 (0.8)	
30 to 34.9	157 (95.7)	7 (4.3)	
25 to 29.9	91 (84.3)	17 (15.7)	
< 25	39 (57.4)	29 (42.6)	

Data are numbers and (row percentages).

3.6 Table S6. Association between mortality and baseline characteristics, multivariate analysis (N=510): sensitivity analysis including patients with missing values

					Univariate				Multivariate			
		N	n	(%)	OR	(95%CI)	*p*	*global p*	aOR	95%CI	*p*	*global p*
**Sex**	**Female**	**252**	**28**	**(11.1%)**	**1.00**	-	-	-	**1.00**	-	-	-
	**Male**	**258**	**34**	**(13.2%)**	**1.21**	**(0.71 – 2.07)**	**0.48**		**0.97**	**(0.44 – 2.14)**	**0.93**	
**Age**	**<45**	**361**	**25**	**(6.9%)**	**1.00**	-	-	-	**1.00**	-	-	-
	**≥45**	**149**	**37**	**(24.8%)**	**4.44**	**(2.56 – 7.70)**	**<0.0001**	-	**8.12**	**(3.52 – 18.7)**	**<0.0001**	
**NEWS2**	**< 7**	**419**	**30**	**(7.2%)**	**1.00**	-	-		**1.00**	-	-	
	**≥ 7**	**65**	**30**	**(46.2%)**	**11.1**	**(6.02 – 20.5)**	**<0.0001**	**<0.0001**	**7.64**	**(3.29 – 17.7)**	**<0.0001**	**<0.0001**
	MV	*26*	*2*	*(7.7%)*	*1.08*	*(0.24-4.79)*	*0.92*		*4.16*	*(0.72-24.0)*	*0.11*	
**Plasma ALT**	**<3 ULN**	***341***	***21***	***(6.2%)***	**1.00**	-	-		**1.00**	-	-	
	**≥ 3 ULN**	***80***	***23***	***(28.8%)***	**6.15**	**(3.19 – 11.8)**	**<0.0001**	**<0.0001**	**2.98**	**(1.19 – 7.42)**	**0.02**	**<0.0001**
	MV	*89*	*18*	*(20.2%)*	*3.86*	*(1.96-7.63)*	*0.0001*		*3.92*	*(1.40-11.0)*	*0.009*	
**KDIGO stage**	**<2**	**442**	**26**	**(5.9%)**	**1.00**	-	-		**1.00**	-	-	
	**≥ 2**	**53**	**28**	**(52.8%)**	**17.9**	**(9.18 – 35.0)**	**<0.0001**	**<0.0001**	**10.30**	**(4.32 – 24.7)**	**<0.0001**	**0.012**
	MV	*15*	*8*	*(53.3%)*	*18.3*	*(6.15-54.3)*	<0.0001		*12.1*	*(2.96-49.1)*	*0.0005*	
**Lassa RT-PCR Ct**	**≥30**	***290***	***8***	**(2.8%)**	**1.00**	-	-		**1.00**	-	-	
	**<30**	***176***	***46***	**(26.1%)**	**12.5**	**(5.72 – 27.2)**	**<0.0001**	**<0.0001**	**7.91**	**(2.90 – 21.5)**	**0.0001**	**0.0003**
	MV	*44*	*8*	*(18.2%)*	*7.83*	*(2.77-22.1)*	*0.0001*		*3.53*	*(0.95-13.2)*	*0.06*	

MV: missing value

3.7 Table S7. Baseline Lassa virus RT-PCR GPC gene Ct value by age, baseline NEWS2, baseline KDIGO grade and baseline plasma ALT value

		Lassa RT-PCR Ct value									
		<25		25-29		30-34		>35		Missing	
		N	(%)	N	(%)	N	(%)	N	(%)	(%)	N
**Age**	< 45 years	40	(11.1)	74	(20.5)	119	(33.0)	100	(27.7)	28	(7.8)
	≥ 45 years	28	(18.8)	34	(22.8)	45	(30.2)	26	(17.4)	16	(10.7)
**NEWS 2**	< 7	36	(8.6)	90	(21.5)	142	(33.9)	112	(26.7)	39	(9.3)
	≥ 7	31	(47.7)	11	(16.9)	13	(20.0)	6	(9.2)	4	(6.2)
**KDIGO grade**	< 2	40	(9.0)	95	(21.5)	152	(34.4)	121	(27.4)	34	(7.7)
	≥ 2	23	(43.4)	13	(24.5)	12	(22.6)	1	(1.9)	4	(7.5)
**Plasma ALT**	< 3 ULN	24	(7.0)	72	(21.1)	128	(37.5)	100	(29.3)	17	(5.0)
	≥ 3 ULN	31	(38.8)	27	(33.8)	11	(13.8)	5	(6.3)	6	(7.5)

N: number; % row percentage

4 Supplementary Figures

4.1 Figure S1. Distribution of the baseline Lassa virus RT-PCR *GPC gene* Ct value in participants included in the analysis (N = 466)

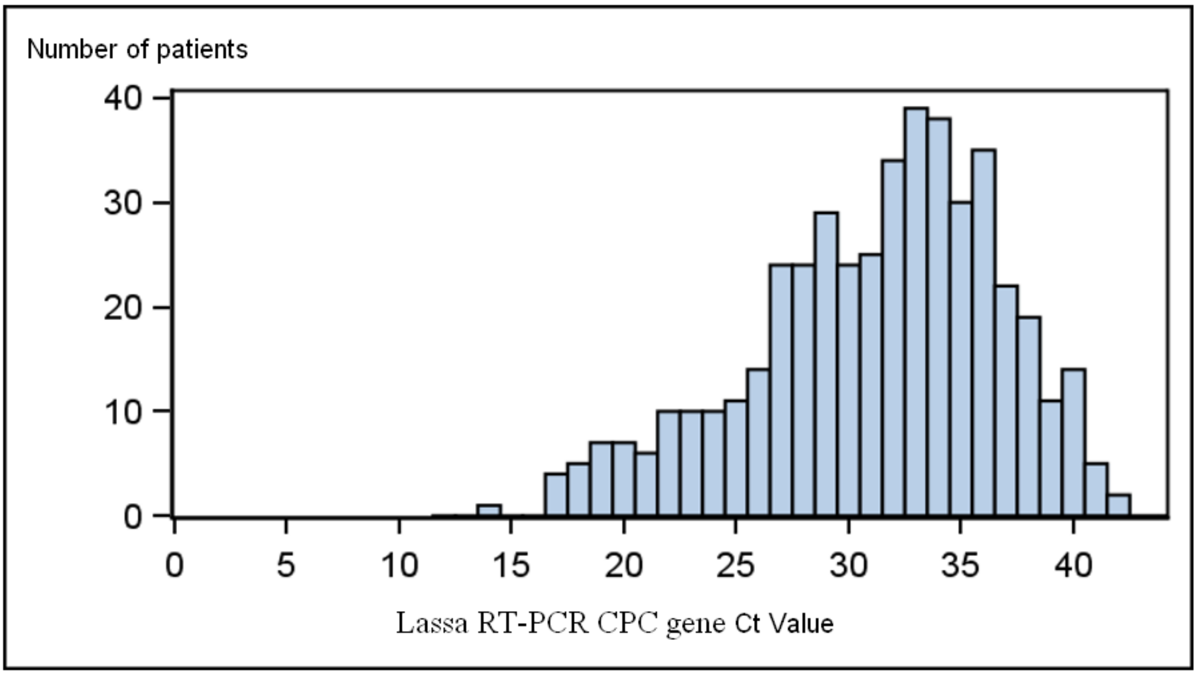

4.2 Figure S2. Lassa virus RT-PCR result at each testing point in Lassa RT-PCR positive participants included in the analysis (N = 510)

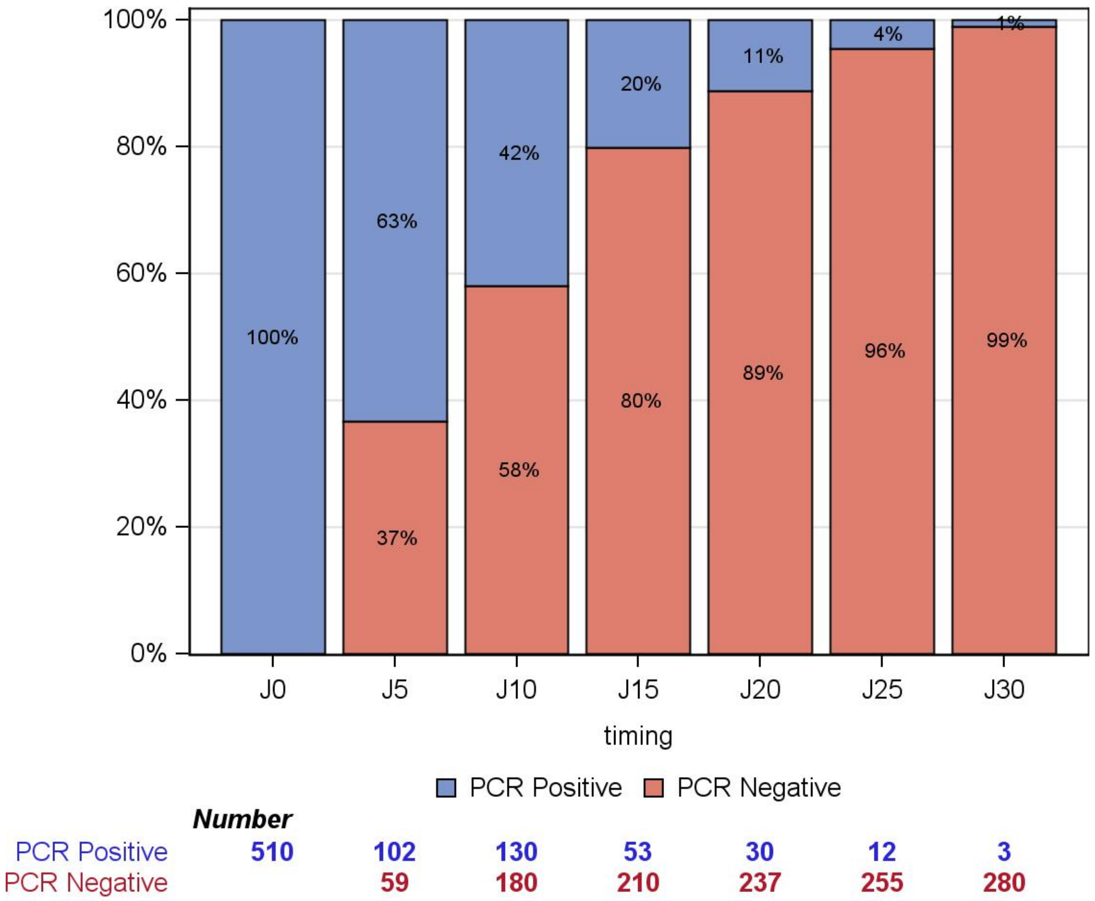

4.3 Figure S3. Kaplan Meier probability of death among Lassa RT-PCR positive participants with a known date of symptom onset (N=505*) [Time from symptoms onset]

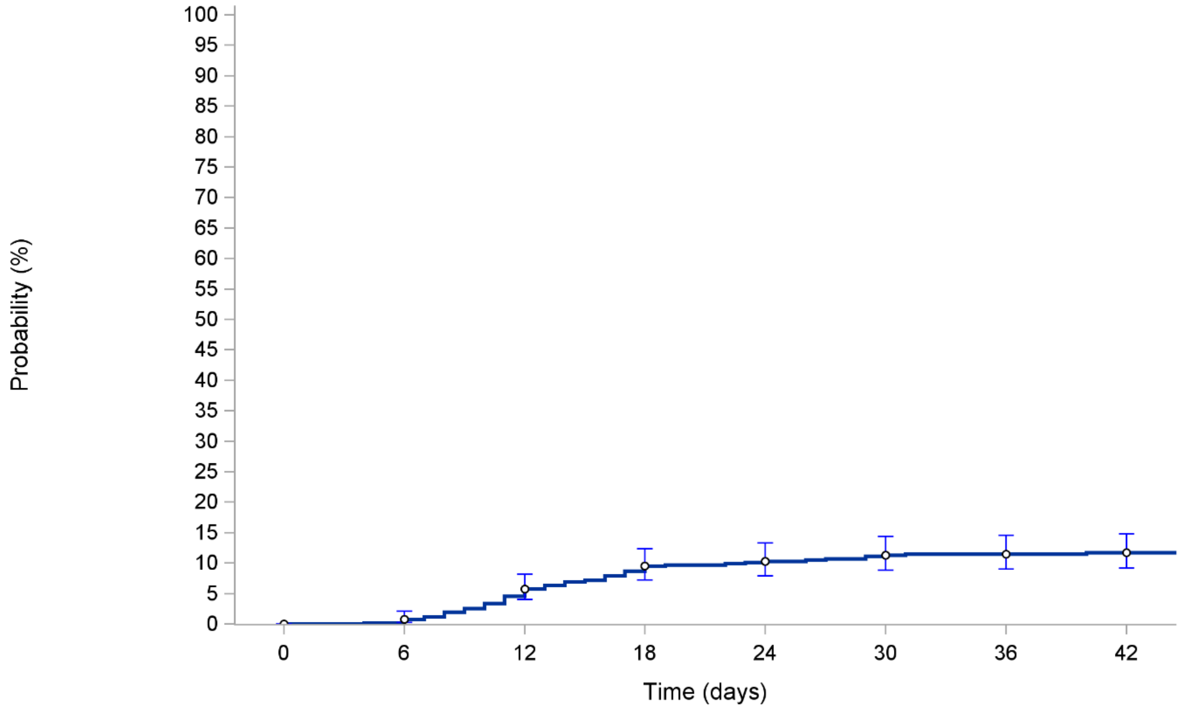

	0	6	12	18	24	30	36	42
Nb of Subjects at Risk	505	501	476	457	453	448	447	446
Nb of events	0	4	29	48	52	57	58	59
Probability of events	0.0	0.8	5.7	9.5	10.3	11.3	11.5	11.7
95% CI of events								
ic	**(0.0-0.0)**	**(0.3-2.1)**	**(4.0-8.2)**	**(7.2-12.4)**	**(7.9-13.3)**	**(8.8-14.4)**	**(9.0-14.6)**	**(9.2-14.8)**

*Five patients had an unknown date of symptoms onset

4.4 Figure S4. Kaplan Meier probability of death among Lassa virus RT-PCR positive participants included in the analysis (N=510) [Time from admission in the Lassa ward]

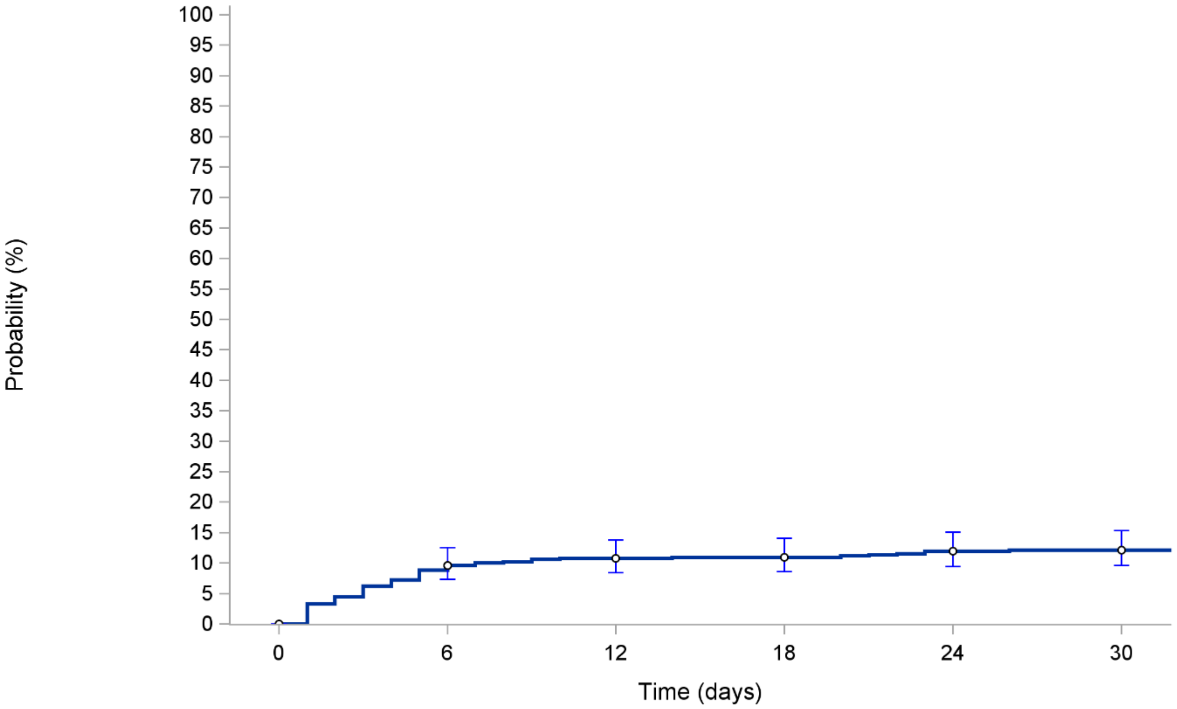

Nb of Subjects at Risk	510	461	455	454	449	448
Nb of events	0	49	55	56	61	62
Probability of events	0.0	9.6	10.8	11.0	12.0	12.2
	95% CI of events					
ic	**(0.0-0.0)**	**(7.3-12.5)**	**(8.4-13.8)**	**(8.6-14.0)**	**(9.4-15.1)**	**(9.6-15.3)**
